# Assessment of Pulmonary Function After Treatment of Scoliosis: Meta-Analysis and Review Article

**DOI:** 10.3390/medicina61071127

**Published:** 2025-06-23

**Authors:** Majdi Hashem

**Affiliations:** Department of Orthopedics, College of Medicine, Imam Mohammad Ibn Saud Islamic University (IMSIU), P.O. Box 90950, Riyadh 11623, Saudi Arabia; mahashem@imamu.edu.sa

**Keywords:** scoliosis, pulmonary function, respiratory outcomes, forced vital capacity (FVC), forced expiratory volume in 1 s (FEV1)

## Abstract

*Background and Objectives:* Pulmonary function is a key outcome in scoliosis management, as both the condition and its treatments can impact respiratory mechanics. This systematic review aimed to assess the effects of scoliosis interventions on pulmonary function, focusing on forced vital capacity (FVC), forced expiratory volume in 1 second (FEV1), and peak expiratory flow (PEF). *Materials and Methods:* A comprehensive literature search was conducted in PubMed, Web of Science, Scopus, and the Cochrane Library to identify studies evaluating pulmonary function before and after scoliosis treatment. Data on respiratory parameters, intervention types, and follow-up periods were extracted. Meta-analyses were performed using standardized mean differences (SMDs) with 95% confidence intervals (CIs). Heterogeneity was assessed using the I^2^ statistic. *Results:* The meta-analysis revealed no significant overall effect of scoliosis interventions on FVC or FEV1. For FVC, the pooled effect size was 0.0126 (95% CI: −0.0161 to 0.0413; *p* = 0.3728), and for FEV1, it was 0.0034 (95% CI: −0.0452 to 0.0519; *p* = 0.8869). Heterogeneity was minimal (I^2^ = 0.0%) for both metrics. Individual studies showed variability: some reported increases in FVC and FEV1 by over 1.5 L, while others observed decreases in percent predicted values and absolute volumes. PEF generally improved, with some interventions showing statistically significant gains (*p* < 0.001). *Conclusions:* Non-invasive rehabilitation methods, such as breathing exercises and aquatic therapy, were associated with more consistent improvements in pulmonary function. In contrast, the effects of surgical interventions were variable and often not statistically significant. These findings suggest a promising role for conservative therapies in enhancing long-term respiratory outcomes in scoliosis patients, though further high-quality research is warranted.

## 1. Introduction

The normal spine appears straight when viewed from the back. Scoliosis occurs when this alignment is disrupted by a curvature, which can involve one or more spinal segments. This curvature is often accompanied by vertebral rotation and wedging. Additionally, scoliosis may result in thoracic prominence or asymmetry of the shoulders and pelvis. Most cases arise in adolescent females without a clear underlying cause, whereas spinal deformities in younger children are more likely to have a discernible origin [1,2].

Pulmonary function testing is widely regarded as a foundational tool for diagnosing various pulmonary diseases. While the methodologies for these tests are well established and commonly used, questions remain about optimal testing procedures, ensuring data accuracy, the selection of appropriate reference values and criteria, and the interpretation of results to support clinical decisions effectively [3,4].

Idiopathic scoliosis, a prevalent condition characterized by lateral displacement and rotation of the vertebral bodies during rapid growth phases, significantly impacts respiratory function. It leads to restrictive lung disease through multiple mechanisms, including reduced lung volumes, displacement of intrathoracic organs, restricted rib movement, and altered respiratory muscle mechanics. Scoliosis also reduces the compliance of both the chest wall and lungs, increasing the effort required for breathing at rest, during physical activity, and while sleeping. In severe cases, complications such as pulmonary hypertension and respiratory failure can arise [5,6].

Previous studies have indicated a relationship between thoracic spinal deformity and pulmonary impairment. Factors such as the severity of the curve, the number of affected vertebrae, the location of the curve, and reduced thoracic kyphosis have all been identified as contributing to pulmonary dysfunction, although the strength of these associations has varied [7].

Severe idiopathic scoliosis can result in respiratory failure, which may require treatment with assisted ventilation. However, the long-term pulmonary outcomes for patients with adolescent idiopathic scoliosis who undergo surgical correction remain unclear. Preoperative pulmonary function test (PFT) values and the use of open anterior surgical approaches account for the largest proportion of variation in PFT results 2 years following surgery. Additionally, thoracoplasty is associated with a clinically significant reduction in predicted pulmonary function at the 2-year mark, though the impact of both factors is relatively modest. These findings may help guide decision making regarding surgical interventions [8,9,10].

This study aimed to systematically assess the effects of scoliosis treatments on pulmonary (respiratory) function, with a focus on both short- and long-term outcomes. I aimed to compare the impact of various treatment modalities, including non-surgical and surgical options, and identify key factors that influenced the improvement or decline in respiratory performance among scoliosis patients.

The objectives of this study included evaluating the effects of exercise-based interventions, such as breathing exercises and conventional exercise therapy, on respiratory function. I also assessed the impact of surgical interventions, particularly spinal fusion, on pulmonary function, analyzing parameters, such as forced vital capacity (FVC), forced expiratory volume in 1 second (FEV1), total lung capacity (TLC), and others. Additionally, I aimed to compare different surgical approaches, such as thoracoscopic versus traditional thoracotomy, to understand their respective effects on pulmonary health. Another objective was to explore the potential benefits of combining breathing exercises with surgery in improving respiratory outcomes for scoliosis patients.

To evaluate these effects, several key factors were assessed, including lung function parameters, such as FVC, FEV1, peak expiratory flow (PEF), TLC, functional residual capacity (FRC), residual volume (RV), and the FEV1/FVC ratio. These factors provided valuable insights into how different treatments affect pulmonary function, guiding clinicians in making informed decisions about managing scoliosis patients’ respiratory health.

## 2. Materials and Methods

This systematic review was conducted in accordance with the Preferred Reporting Items for Systematic Reviews and Meta-Analyses (PRISMA) 2020 guidelines. The PRISMA checklist is provided in the Supplementary Materials.

### 2.1. Search Strategy

A comprehensive search strategy was employed to identify relevant studies on pulmonary function following scoliosis treatment. The databases searched included PubMed, WOS, Scopus, and Cochrane Library. Keywords and medical subject headings (MeSH) were combined using Boolean operators, focusing on terms, such as “scoliosis”, “pulmonary function”, “respiratory outcomes”, “forced vital capacity (FVC)”, “forced expiratory volume (FEV1)”, and “postoperative recovery.” Searches were restricted to studies published in English without time restrictions to ensure a thorough inclusion of the relevant literature. Additional articles were identified through manual reference checks of selected studies and systematic reviews, along with Boolean operators (AND, OR) to refine the search. The search included studies from the last 20 years published in English, and the manual screening of references from relevant articles was performed to identify additional studies not captured by database searches.

### 2.2. Study Selection

Eligible studies included peer-reviewed articles that evaluated pulmonary function in patients undergoing scoliosis treatment. Inclusion criteria were studies reporting pulmonary function outcomes before and after treatment, such as FVC, FEV1, and PEF, using validated respiratory tests; I included randomized and non-randomized studies. Exclusion criteria included studies lacking quantitative respiratory data, case reports, or studies focusing on non-surgical treatments or unrelated pulmonary conditions. Two independent reviewers screened titles and abstracts, followed by a full-text review for eligibility, resolving any discrepancies through discussion or consultation with a third reviewer.

### 2.3. Data Extraction

The data extraction was conducted by two independent investigators. The extracted information encompassed study characteristics, participant details, intervention specifics, and outcome measures.

### 2.4. Quality Assessment

The quality of the studies was appraised independently by two investigators. We assessed the risk of bias in included studies using the Cochrane risk-of-bias tool for RCTs [11]. This tool is transparent and reproducible and facilitates evidence-based decision making by identifying potential biases in study design and reporting. Moreover, the quality of non-randomized trials was evaluated using the Newcastle–Ottawa Scale (NOS).

### 2.5. Data Analysis

Statistical analyses were performed using R software 4.2.2. Continuous outcomes were assessed through mean differences [SE] with 95% CI. Heterogeneity with *p*-value 0.05 was determined using the Cochrane Q *p*-value and I^2^ statistic, where values below 50% indicated low heterogeneity. Both random-effects and common-effect models were applied, and publication bias was examined using funnel plots and Egger’s regression test.

## 3. Results

### 3.1. Study Selection and Characteristics

**Figure 1:**
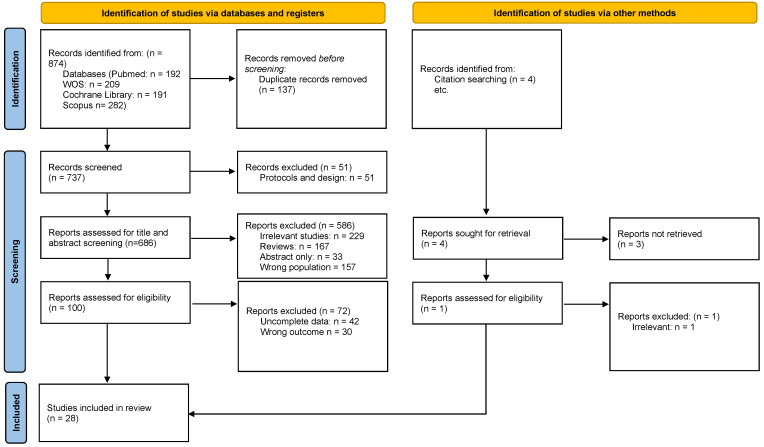
PRISMA flow chart.

The literature search yielded 874 records, of which 137 were duplicates. The remaining 737 articles were then evaluated based on title and abstract, with 619 deemed irrelevant and removed. The remaining 100 articles were evaluated using the full text for eligibility, and 72 were excluded. The overall count of incorporated studies was 28 [12,13,14,15,16,17,18,19,20,21,22,23,24,25,26,27,28,29,30,31,32,33,34,35,36,37,38,39]. Figure 1 outlines the schematic flow of the studies’ identification and inclusion processes.

### 3.2. Demographic Characteristics of Included Studies

This systematic review included a diverse range of studies to evaluate the impact of various treatments on pulmonary function in patients with scoliosis. The studies spanned multiple designs, including RCTs, retrospective and prospective studies, and cohort designs, conducted in different countries, such as the USA, India, China, Turkey, and Poland. Interventions ranged from surgical techniques such as spinal fusion and thoracoscopic approaches to conservative treatments such as breathing exercises, aquatic programs, and rehabilitation protocols. Participants’ ages varied widely, with many studies focusing on adolescents and young adults, while gender distributions also showed variation, with some studies having predominantly female or male participants (Table 1).

### 3.3. FVC Outcomes

The studies reviewed indicated varying outcomes in FVC following different surgical interventions. Kumar (2024) [12] reported increases of 1.59 L and 1.81 L in FVC from baseline, suggesting significant functional improvement following surgery. In contrast, Mallepally et al. (2018) [28] observed a reduction in vital capacity from 90.6% to 86.2% 2 years after operation, indicating a decline over time. Rafferty et al. (2020) [32] found a notable difference between AIS cohort groups, with a reduction in FVC from 2.6 ± 0.5 L to 3.3 ± 0.5 L (*p* < 0.001), highlighting varying surgical impacts on lung function.

Further studies, such as those by Ogonowska-Slodownik et al. (2021) [30] and Küçük et al. (2024) [13], showed divergent trends in FVC outcomes. Ogonowska-Slodownik et al. (2021) [30] reported a slight increase from 3.54 L to 3.61 L in one group but a decrease from 3.51 L to 3.32 L in another, indicating potential differences in surgical techniques or patient responses. Küçük et al. (2024) [13] observed a modest improvement from 2.74  ±  1.07 L to 2.9  ±  1.27 L, suggesting some recovery of lung function, though not to baseline levels.

Studies by Lonner et al. (2009) [25] and Yaszay et al. (2019) [35] provided insights into longer-term outcomes. Lonner et al. (2009) [25] found minor changes in FVC, with preoperative values at 2.96 L and values at follow-up at 2.70 L (change −0.26 L, *p* < 0.001) in one group and minimal change in another group (preoperative: 2.88 L; follow-up: 2.90 L; change +0.02 L, NS). This suggested that while some surgical corrections might lead to slight decreases in lung function, others did not significantly alter the preoperative FVC. Conversely, Yaszay et al. (2019) [35] reported a significant increase from 2.98  ±  0.7 L preoperatively to 3.15  ±  0.7 L at 5 years (*p* = 0.008), demonstrating long-term functional improvement in selected patients (Table 2).

### 3.4. FEV1 Outcomes

The changes in FEV1 following surgical interventions across several studies indicated varied outcomes. Several studies reported a significant improvement in FEV1 after surgery, including Kumar (2024 A) and Kumar (2024 B) [12], where FEV1 increased by 1.48 L and 1.66 L, respectively. Additionally, Ogonowska-Slodownik et al. (2021 A) [30] observed an increase in FEV1 from 2.88 L to 3.05 L, suggesting a positive surgical effect on pulmonary function.

Conversely, other studies presented more modest improvements or even declines. For example, the study by Lonner et al. (2009 A) [25] showed a slight decrease of 0.12 L in FEV1 (*p* = 0.004), while the study by Yu et al. (2017) [36] did not report significant changes. Furthermore, the research by Yaszay et al. (2019) [35] showed an increase from 2.48 L to 2.58 L over 5 years, though the change was not statistically significant (*p* = 0.10).

Some studies, such as those by Dogar et al. (2021 A and B) [19], indicated fluctuations, with changes in FEV1 at different follow-up times. Specifically, Dogar et al. (2021 A) [19] reported an increase from 2.47 L to 2.93 L 6 months following surgery, while Dogar et al. (2021 B) [19] reported a small improvement from 2.15 L to 2.44 L at the same interval. These variations could be attributed to different surgical techniques, patient populations, and follow-up durations.

Several studies also indicated minimal to no changes in FEV1. For example, the study by Newton et al. (2013 B) [29] showed a slight decrease in FEV1 from 2.4 L to 2.3 L (*p* = −0.2 ± 0.4), which suggested that some patients may not have experienced significant improvements in lung function after surgery. Similarly, Kim et al. (2018 A) [14] observed a decrease of −0.21 L (*p* = 0.015) in FEV1 2 years following surgery, though this change was statistically significant.

In contrast, some studies such as those by Yildirim et al. (2019 A and B) [38] reported substantial improvements in FEV1, with changes of +7.35% and +2.92%, respectively. These findings suggested that certain interventions might be more effective in enhancing pulmonary function (Table 2).

### 3.5. PEF Outcomes

The discussion on PEF highlights a mix of reported improvements and data gaps across various studies. Notable findings included Kumar (2024) [12], which demonstrated significant increases in PEF by 3.77 L/s and 4.11 L/s in two study arms, reflecting consistent positive outcomes. Similarly, Küçük et al. (2024) [13] showed substantial improvements, with PEF rising from 243.75 ± 69.77 L/min to 289.25 ± 83.92 L/min in one arm (*p* = 0.001) and from 237.25 ± 87.68 L/min to 276.5 ± 103.68 L/min in another. Amăricăi et al. (2019) [17] reported a modest increase from 5.12 to 5.22 L/min, while Yildirim et al. (2019) [38] observed significant percentage gains in two groups, with changes of +13.32% and +4.67% (*p* < 0.0001). Additionally, Akazawa et al. (2018) [16] noted a rise in PEF from 3.67 ± 1.51 L/s to 4.38 ± 1.18 L/s, with a change of +0.71 L/s (*p* = 0.029). However, many studies, including those by Mallepally et al. (2018), Rafferty et al. (2020), and Newton et al. (2013) [28,29,32], did not report PEF data, highlighting gaps in the literature. Some studies, such as the one by Yuan et al. (2005) [39], acknowledged the PEF but did not include measurements, underscoring the variability in data reporting (Table 2).

### 3.6. Other Outcomes

The analysis of other respiratory outcomes across various studies revealed a diverse range of results, emphasizing both improvements and challenges in pulmonary function measures following intervention. Kumar (2024) [12] demonstrated significant enhancements in the FEV1/FVC ratio, with changes of 85.12% and 81.32% across two study arms, coupled with increased vital capacity (VC) by 1.78 L and 2.06 L, respectively. Similarly, Ogonowska-Slodownik et al. (2021) [30] observed an increase in FEV1%VC and predicted FEV1%VC in one arm, though the other arm showed declines in these metrics, highlighting variability. Küçük et al. (2024) [13] reported a modest yet significant improvement in FEV1 and FEV1/FVC, affirming positive pulmonary adaptations.

On the other hand, certain studies reported stable or even declining outcomes. Lonner et al. (2009) [25] observed no significant change in total lung capacity (TLC) in one arm, while another arm showed a significant increase of 0.44 L (*p* < 0.001), underlining nuanced results. Similarly, Yaszay et al. (2019) [35] found a significant reduction in the predicted FVC over a 5-year follow-up, suggesting a potential long-term challenge in maintaining pulmonary function. Kim et al. (2018) [14] also observed declines in predictive FVC values postoperatively in two arms, with statistically significant differences.

In some cases, outcomes were mixed or minimal. Studies such as the one by Mallepally et al. (2018) [28] showed minor improvements in TLC and significant gains in subjective scores such as the SRS-30 over 2 years. Akazawa et al. (2018) [16] reported significant improvements in %FEV1 and other flow measures, while Newton et al. (2013) [29] found minimal to no changes in the predicted TLC across arms, with one arm showing a notable decrease of 15%. Yildirim et al. (2019) [38] highlighted significant gains in FEF25–75%, with improvements ranging from 6% to over 11% across two groups.

Lung volume measures, such as those explored by Zhou et al. (2022) and Yu et al. (2017) [36,37], provided additional insights. Zhou et al. documented increases in total lung volume and specific changes in concave and convex lung volumes at a 5-year follow-up. In contrast, Yu et al. reported significant increases in left lung volume but no significant changes in right lung volume (Table 2).

### 3.7. Outcomes According to Intervention Type

#### 3.7.1. Breathing and Exercise-Based Interventions

Several studies on breathing and exercise interventions have shown improvements in pulmonary function. Kumar (2024) [12] demonstrated that both breathing exercises and conventional exercise therapy led to increases in FVC, FEV1, and PEF, with conventional exercise proving slightly more effective. Ogonowska-Slodownik et al. (2021) [30] found that aquatic breathing improved lung function, whereas corrective swimming had a negative effect. Küçük et al. (2024) [13] noted that spinal mobilization combined with core stabilization exercises significantly improved FVC, FEV1, and PEF, whereas core stabilization alone had a moderate impact. Amăricăi et al. (2019) [17] also showed that a rehabilitation program led to improvements in FVC, FEV1, and PEF, although the FEV1/FVC ratio showed only slight gains. These findings indicated that while exercise-based therapies were beneficial for lung function, combining different modalities, such as spinal mobilization or aquatic breathing, yielded better outcomes (Table 2).

#### 3.7.2. Surgical Interventions

Surgical interventions for scoliosis, particularly spinal fusion, have had varied effects on pulmonary function. Mallepally et al. (2018) [28] reported that posterior spinal fusion resulted in a slight decline in FVC and FEV1 but that TLC improved, suggesting that lung capacity might be preserved despite reduced airflow. Rafferty et al. (2020) [32] found that surgery improved FVC and FEV1 in AIS patients, while Yaszay et al. (2019) [35] observed moderate improvement in FVC and FEV1 following surgery but a decline in the predicted FVC%. Ran et al. (2014) [31] highlighted that a combined thoracoscopic anterior release and posterior correction improved FVC and TLC while reducing FRC and RV. Yu et al. (2017) [36] also found that thoracoscopic anterior spinal fusion led to significant improvements in TLC. These findings suggested that surgical interventions can improve lung function, although the effects on predicted lung capacity vary, with thoracoscopic approaches showing a better result (Table 2).

### 3.8. Comparing Surgical Approaches

Comparing different surgical techniques revealed varying impacts on lung function. Lonner et al. (2009) [25] observed that thoracotomy resulted in decreased FVC and FEV1, while thoracoscopic surgery showed minimal changes in FVC and FEV1 and an improvement in TLC. Faro et al. (2005) [20] found that both thoracoscopic and open thoracotomy surgeries caused initial declines in FVC and FEV1 but that recovery occurred over time. These results suggested that thoracoscopic surgery, which is less invasive, led to more favorable outcomes, particularly in terms of lung capacity (Table 2).

### 3.9. Scoliosis Surgery With or Without Breathing Exercises

Dogar et al. (2021) [19] studied the combination of scoliosis surgery and breathing exercises, finding that while surgery alone led to a decline in lung function initially, the addition of breathing exercises facilitated recovery. Both groups experienced some improvement over time, but the group with breathing exercises showed more favorable results. This suggested that integrating breathing exercises with scoliosis surgery can enhance recovery and improve pulmonary function in the long term (Table 2).

### 3.10. Meta-Analysis of FVC Change

The meta-analysis examining the change in FVC across 25 studies reveals a very small, statistically insignificant pooled effect size. The random-effects model yields an SMD of 0.0126 with a 95% confidence interval of [−0.0161; 0.0413] and a *p*-value of 0.3728, indicating that there is no significant overall effect. Additionally, heterogeneity between studies is minimal, with an I^2^ statistic of 0.0%, suggesting that the studies are consistent in their findings. The tau^2^ value is 0, further confirming the lack of substantial variation between study effects. While some individual studies, such as Kumar (2024 A) [12], show larger effect sizes, they have wide confidence intervals, and others show negligible effects. The largest contributor to the analysis, Faro et al. (2005 A) [20], has a high weight (65.5%) but still produces a near-zero effect size. In conclusion, the results of this meta-analysis suggest that the interventions tested have a negligible impact on FVC, and the variations seen in individual studies are likely due to random fluctuations rather than a consistent treatment effect.

**Figure 2:**
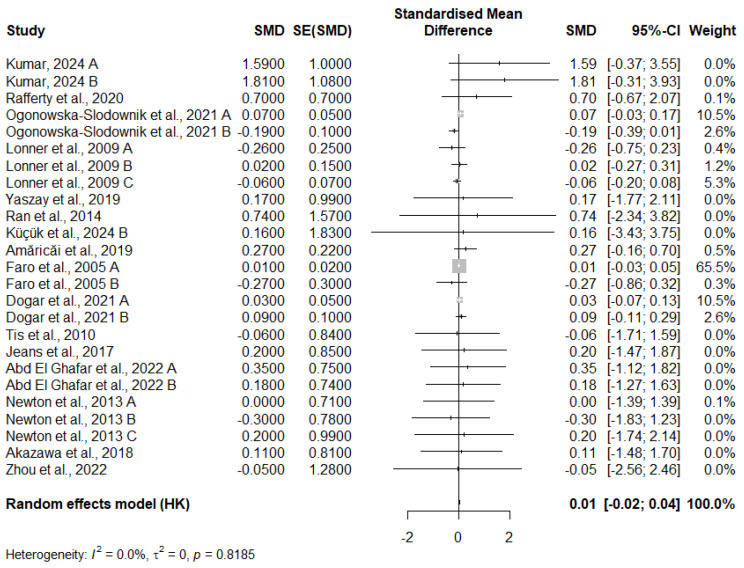
Meta-analysis of FVC change [12,13,15,16,17,19,20,24,25,29,30,31,33,35,37].

Notably, several studies contributed 0% weight to the pooled estimate. This occurred primarily because those studies had either extremely wide confidence intervals, high standard errors, or small sample sizes—factors that reduce their statistical contribution under the inverse-variance method. Overall, this analysis suggests that the intervention has no meaningful effect on FVC, and the findings are robust due to the absence of statistical heterogeneity (Figure 2).

### 3.11. Meta-Analysis of FEV1 Change

The results of the meta-analysis on the SMD for FEV1 changes across 25 studies show that the overall effect size is 0.0034 with a 95% CI of [−0.0452; 0.0519]. This indicates a very small and practically insignificant effect on FEV1. The *p*-value of 0.8869 suggests that the observed effect is not statistically significant, as it is well above the conventional threshold of 0.05.

**Figure 3:**
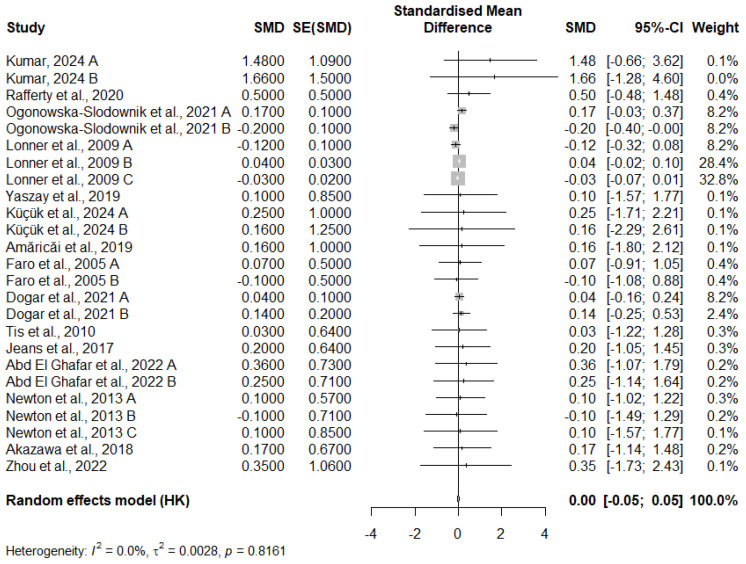
Meta-analysis of FEV1 change [12,13,15,16,17,19,20,24,25,30,32,35].

The I^2^ value is 0.0%, indicating minimal heterogeneity between the studies, meaning that the results from these studies are very consistent. The tau^2^ value is very low (0.0028), with a tau of 0.0527, further confirming the low variability in the effect sizes across the studies. Additionally, the Q statistic (17.72) and the associated *p*-value (0.8161) indicate no significant heterogeneity, suggesting that the results of the individual studies are homogeneous (Figure 3).

### 3.12. Publication Bias

#### 3.12.1. FVC Change

The linear regression test for funnel plot asymmetry (commonly referred to as Egger’s test) was conducted to assess publication bias in the meta-analysis of FVC change. The test yielded a t-value of 1.05, with 23 degrees of freedom, and a *p*-value of 0.3064. This result is not statistically significant, indicating no evidence of publication bias.

**Figure 4:**
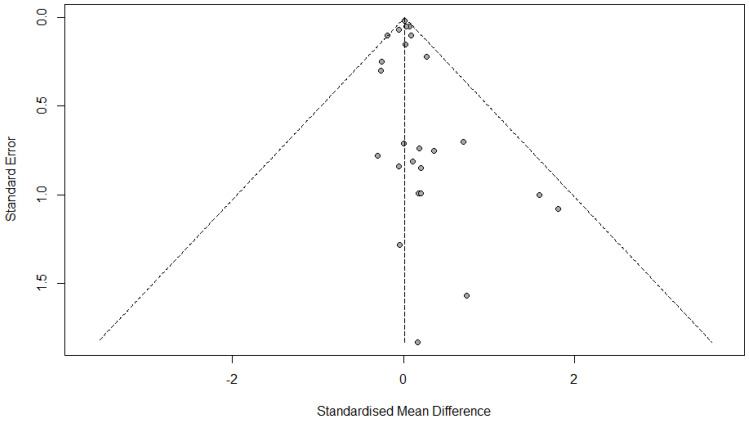
Funnel plot of FVC change.

The bias estimate was 0.2091, with a standard error of 0.1999, which further supports the lack of asymmetry in the funnel plot. A non-zero intercept might suggest small-study effects (e.g., publication bias), but since the *p*-value is greater than the typical significance threshold (e.g., 0.05), we cannot reject the null hypothesis of symmetry (Figure 4).

#### 3.12.2. FEV1 Change

**Figure 5:**
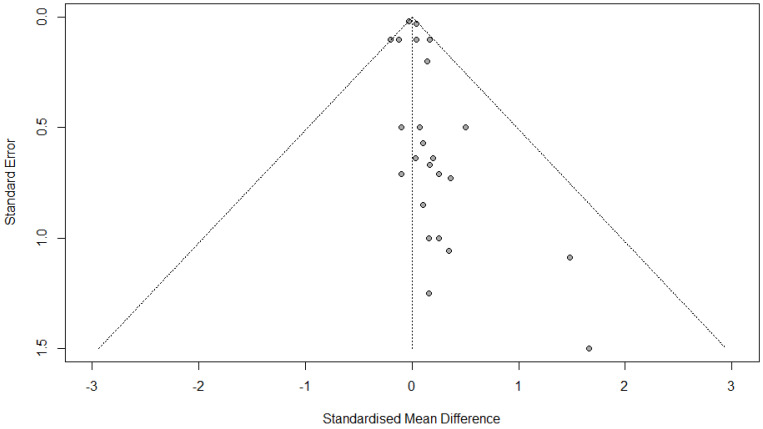
Funnel plot of FEV1 change.

The results of the linear regression test for funnel plot asymmetry suggest a potential, but not strong, indication of publication bias. The *p*-value of 0.0802, which is above the typical 0.05 threshold for statistical significance, means there is no clear evidence of funnel plot asymmetry, which is commonly associated with publication bias. However, the *p*-value is close to the threshold, indicating some possibility of bias. The bias estimate of 0.3418 with a standard error of 0.1868 points to a slight asymmetry in the funnel plot, suggesting that smaller studies or those with less significant results may be underrepresented. The tau^2^ value of 0.6727 indicates some residual heterogeneity in the data, which is not directly linked to publication bias but shows variability between studies. Overall, while the test does not provide strong evidence for publication bias, there is a minor suggestion of bias, but it is not substantial enough to be considered a major concern in this meta-analysis (Figure 5).

#### 3.12.3. Quality Assessment

**Figure 6:**
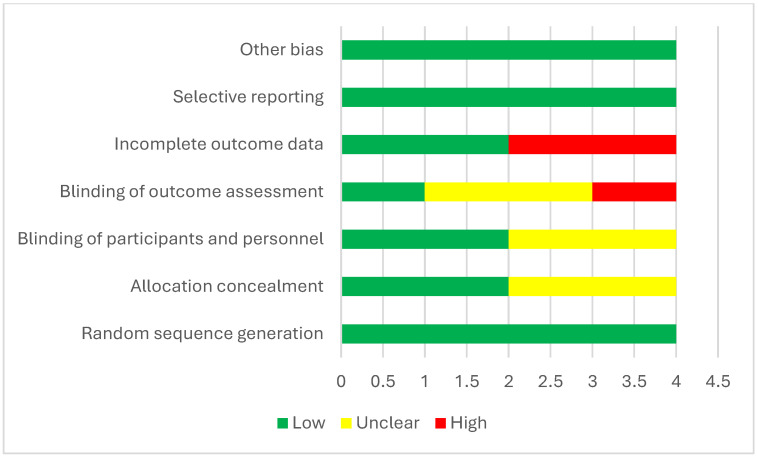
Cochrane risk-of-bias assessment.

Table 3 evaluates the risk of bias across several methodological criteria. Küçük et al. (2024) [13] showed the lowest risk, with “Low” ratings in all domains, indicating a robust methodology. Doger et al. (2021) and Yildirim et al. (2019) [19,38] had “Unclear” ratings for allocation concealment and blinding, suggesting moderate concerns. Abdel Ghafar et al. (2022) [15] exhibited the highest risk due to “High” ratings in blinding and incomplete outcome data, raising significant reliability concerns (Figure 6).

Table 4 evaluates the methodological quality of studies based on Selection, Comparability, and Exposure/Outcome domains, with an overall star rating summarizing their rigor. Studies such as the ones by Lonner et al. (2009), Newton et al. (2013), Kim et al. (2008), and Grabala et al. (2023) [14,22,25,29] scored the maximum eight stars, demonstrating robust methodologies across all domains. High Selection scores (e.g., four stars) indicated rigorous participant selection, as seen in Lonner et al. (2009) and Newton et al. (2013) [25,29], while most studies achieved two stars in Comparability, reflecting adequate control of confounders. In the Exposure/Outcome domain, studies such as Rafferty et al. (2021) and Tis et al. (2010) [32,33] excelled, with three stars for precise outcome measurement. Moderate-quality studies, with six or seven stars (e.g., Byun et al. (2020), Vitale et al. (2008), and Zhou et al. (2022) [18,34,37] showed some limitations in specific domains. Lower-rated studies, such as those by Hu et al. (2015) and Amăricăi et al. (2019) [17,23] scored only four or five stars, reflecting notable weaknesses in Selection or Exposure/Outcome.

## 4. Discussion

In this systematic review, I assessed various studies exploring different treatments and their effects on pulmonary function, as measured by FVC, FEV1, PEF, and other outcomes. The reviewed treatments included different exercise protocols, surgeries, and rehabilitation programs aimed at improving respiratory function in patients with conditions such as scoliosis, spinal deformities, and other respiratory impairments.

For example, Kumar (2024) [12] conducted one study comparing different exercise protocols. A breathing exercise protocol significantly improved pulmonary function, with FVC increasing by 1.59 L, FEV1 by 1.48 L, and PEF by 3.77 L/s. Additionally, the study found that the FEV1/FVC ratio changed by 85.12% and the VC improved by 1.78 L. On the other hand, conventional exercise therapy showed similarly promising results, with FVC increasing by 1.81 L, FEV1 by 1.66 L, and PEF by 4.11 L/s. The FEV1/FVC ratio improved by 81.32% and the VC increased by 2.06 L. These results suggested that both exercise regimens were effective in enhancing pulmonary function, with the conventional therapy showing slightly superior improvements in most parameters.

In contrast, studies such as those by Mallepally et al. (2018) and Lonner et al. (2009) [25,28] focused on surgical interventions. Mallepally’s study examined posterior spinal fusion, noting a slight decline in FVC and FEV1 from before surgery to after surgery over a 2-year follow-up. While the FVC decreased from 90.6 L to 86.2 L and the FEV1 from 86.67 L to 85 L, other outcomes such as the TLC showed improvements. Similarly, Lonner et al. (2009) [25] compared thoracotomy, thoracoscopic, and thoracoabdominal surgeries. Their findings indicated that thoracotomy led to significant decreases in FVC and FEV1, while thoracoscopic surgery led to minimal changes and thoracoabdominal surgery to no significant changes in these measures.

Other studies, such as those by Ogonowska-Slodownik et al. (2021A and B) [30], examined the effects of aquatic breathing programs and corrective swimming. Their findings were mixed. In Study A, the aquatic breathing program increased the FVC from 3.54 L to 3.61 L and the FEV1 from 2.88 L to 3.05 L, while in Study B, corrective swimming led to decreases in both the FVC and FEV1, indicating that different types of aquatic exercise might have varying impacts on pulmonary function. These results suggested that while certain breathing exercises may provide benefits, other forms of physical activity, such as swimming, might not always yield the same improvements.

The study by Yaszay et al. (2019) [35] further investigated the long-term outcomes of surgery, reporting a significant increase in FVC from before surgery to the 5-year follow-up. However, the FEV1 showed no significant change, highlighting that while surgical interventions might improve lung volume, they might not necessarily enhance airflow function to the same extent. Similarly, Ran et al. (2014) [31] observed improvements in FVC after anterior thoracoscopic release and posterior correction, but changes in FRC and TLC were also notable, indicating the complex nature of post-surgical pulmonary recovery.

Küçük et al. (2024) [13] contributed valuable insights to the effects of spinal mobilization and core stabilization exercises on pulmonary outcomes. In their study, FEV1 improved following treatment, while PEF also showed significant improvements, suggesting that a combination of spinal stabilization and respiratory exercises could lead to better pulmonary outcomes, especially for individuals with limited lung function before treatment.

The studies examined showed varied results in the effects of different interventions on lung function, particularly focusing on FVC, FEV1, PEF, and other respiratory parameters. Kumar (2024) [12] reported significant improvements in both FVC and FEV1 with breathing exercise protocols and conventional exercise therapy, with the breathing exercise protocol showing slightly less improvement in the FEV1/FVC ratio but a greater increase in the PEF. Conversely, Mallepally et al. (2018) and Rafferty et al. (2020) [28,32] found that surgical interventions, such as posterior instrumentation and fusion, showed small decreases in lung function over time, though Mallepally’s study noted a slight improvement in TLC. Other studies, such as the one by Ogonowska-Slodownik et al. (2021) [30], examined aquatic breathing programs and corrective swimming, with the aquatic breathing program showing mild increases in lung function and the corrective swimming regimen leading to small declines. Studies such as one by Küçük et al. (2024) [13] that focused on core stabilization exercises showed improvements in FEV1 and PEF following an intervention, highlighting the benefit of rehabilitation protocols alongside medical treatments. In contrast, studies focused on thoracoscopic and other surgical techniques, such as those by Lonner et al. (2009) and Yaszay et al. (2019) [25,35], found no significant improvements in lung function, with some patients experiencing declines or negligible changes.

While various therapeutic and surgical interventions were tested, the effects on lung function varied significantly across studies. Rehabilitation-focused interventions such as breathing exercises, spinal mobilization, and core stabilization exercises tended to show the most consistent improvements in lung function parameters. Surgical interventions, particularly those involving thoracic procedures, demonstrated more mixed results, with some studies indicating declines or minimal changes. This suggested that non-invasive rehabilitation therapies may offer a more predictable and beneficial impact on lung function in patients, though further research is needed to clarify long-term outcomes, particularly for surgical interventions.

Overall, the reviewed studies provided a broad perspective on the diverse approaches to improving pulmonary function, with both exercise-based therapies and surgical interventions demonstrating varying degrees of success. While breathing exercises, particularly those in combination with other rehabilitation programs, showed consistent improvements in respiratory outcomes, surgical treatments, like spinal surgeries, had more mixed results. The findings underscored the need for further research to refine these treatments and understand their long-term effects on patients with respiratory and spinal conditions.

### 4.1. Strengths

This systematic review and meta-analysis had several notable strengths, including the diversity of treatment approaches evaluated, such as exercise-based therapies, surgeries, and aquatic breathing programs. These varied modalities provided a comprehensive perspective on the different strategies for improving pulmonary function. Additionally, several studies reported significant improvements in key pulmonary measures such as FVC, FEV1, and PEF, demonstrating the effectiveness of these treatments. Long-term follow-up in studies such as that of Yaszay et al. (2019) [35], which tracked patients for up to 5 years, offered valuable insights into the sustainability of treatment effects. The inclusion of different patient groups also enhanced the generalizability of the findings to a broad clinical context.

### 4.2. Limitations

Despite its strengths, this review also had several limitations. The treatment protocols varied considerably, including differences in exercise intensity, duration, and type, which contributed to inconsistencies in the results. Some studies had short follow-up periods, which limited the understanding of the long-term effects of the treatments, especially for surgical interventions. Moreover, many studies did not consistently account for confounding factors such as age, gender, or baseline health status, potentially impacting the outcomes.

### 4.3. Recommended Future Research

To address these limitations, future research should aim for larger, more diverse sample sizes to increase the statistical power and enhance the generalizability of the findings. Standardized measurement tools and protocols are necessary to ensure consistency across studies, allowing for better comparisons of results. Longer follow-up periods are also crucial, particularly for surgical treatments, to assess the sustainability of improvements in pulmonary function. Moreover, exploring the combined effects of various treatment modalities, such as pairing exercise therapies with surgical interventions, could provide insights into the most effective multi-faceted approaches. Finally, future studies should focus on specific patient populations, such as children with scoliosis or adults with respiratory conditions, to develop more targeted and effective treatments.

## 5. Conclusions

Non-invasive rehabilitation methods, such as breathing exercises, core stabilization, and aquatic therapy, consistently improve lung function parameters like FVC, FEV1, and PEF. In contrast, surgical interventions, especially thoracic and spinal procedures, show variable and less predictable outcomes, with some studies reporting no improvement or slight declines. While surgery may be necessary in specific cases, rehabilitation appears more effective for long-term respiratory improvement.

## Figures and Tables

**Table 1:** Demographic characteristics of included studies.

Studies	Study Design	Treatment	Country of the Patients	Number of Participants	Age	Gender (Female/Male %)
Kumar, 2024 A [12]	Pre and post test	Breathing exercise protocol	India	18	10 and 15 years	47.2/52.8%
Kumar, 2024 B [12]	Pre and post test	Conventional exercise therapy	India	18	10 and 15 years	
Mallepally et al., 2018 [28]	Retrospective, observational study	Posterior instrumentation and posterior spinal fusion	India	41	11 to 19 years	61/39%
Rafferty et al., 2020 [32]	Retrospective, case control study	Surgery	Ireland	20	18 (2)	_
Ogonowska-Slodownik et al., 2021 A [30]	Pre and post test	Aquatic Breathing Program	Poland	13	14.2 (1.4)	_
Ogonowska-Slodownik et al., 2021 B [30]	Pre and post test	Corrective swimming	Poland	10	14.2 (1.4)	_
Lonner et al., 2009 A [25]	Prospective chart review	Thoracotomy	USA	68	14.3 (10–21)	89.7/10.3
Lonner et al., 2009 B [25]	Prospective chart review	Thoracoscopic	USA	44	13.9 (10–18)	88.6/11.4%
Lonner et al., 2009 C [25]	Prospective chart review	Thoracoabdominal	USA	19	14.8 (13–18)	94.7/5.3%
Yaszay et al., 2019 [35]	Retrospective study	Surgery	USA	262	10–21 years	82.8/17.2%
Ran et al., 2014 [31]	Retrospective study	Anterior thoracoscopic release combined with posterior correction	China	28	16.3 (12–27)	57.1/42.9%
Yu et al., 2017 [36]	Cohort	Thoracoscopic anterior spinal fusion	_	23	15.7 (3.1)	100/0%
Küçük et al., 2024 A [13]	RCT	spinal mobilisation followed by core stabilisation exercises	Turkey	20	12.9 ± 1.8	60/40%
Küçük et al., 2024 B [13]	RCT	Core stabilisation exercises	Turkey	20	12.85 ± 1.81	65/35%
Amăricăi et al., 2019 [17]	Non-randomized	Rehabilitation programme	Romania	40	13.10 ± 2.8	60/40%
Faro et al., 2005 A [20]	Prospective study	Thoracoscopic	USA	31	13.3 (1.5)	96.8/3.2%
Faro et al., 2005 B [20]	Prospective study	Open thoracotomy for anterior spinal fusion	USA	23	14.8 (2.3)	82.6/17.4%
Dogar et al., 2021 A [19]	Randomized trial	Scoliosis surgery and diaphragmatic breathing and pursed lip exercises	Turkey	15	16.3 ± 3.48	_
Dogar et al., 2021 B [19]	Randomized trial	Scoliosis surgery	Turkey	15		_
Hu et al., 2015 [23]	Retrospective study	Posterior temporary internal distraction correction and definitive posterior spinal correction with posterior pedicle screw instrumentation	China	11	mean: 17.8 (15–23)	72.7/27.8%
Tis et al., 2010 [33]	Prospective study	Open instrumented anterior spinal fusion	_	85	_	_
Fu et al., 2015 [21]	Prospective study	Posterior spinal fusion surgery	China	30	10–16 years	70/30%
Jeans et al., 2017 [24]	Prospective study	Spinal fusion	USA	37	14.5 (11.3–18.7)	81/19%
Min et al., 2012 [27]	Prospective study	Anterior short correction	Switzerland	62	15.2 (SD 2.5)	_
Abd El Ghafar et al., 2022 A [15]	RCT	Hippotherapy combined with Schroth exercises	KSA	22	14.68 ± 1.72	59/41%
Abd El Ghafar et al., 2022 B [15]	RCT	Schroth exercises	KSA	23	15.04 ± 1.66	65.2/34.8%
Vitale et al., 2008 [34]	Retrospective study	Spinal fusion	USA	21	12.6 (3.5)	57.1/42.9%
Laurentowska et al., 2009 [26]	Cohort	Cotrel-Dubousset plus Rehabilitation program	Poland	16	16.6 (1.5)	_
Yuan et al., 2005 [39]	Prospective study	Surgery	USA	24	12.7 (2.7)	58.3/41.7%
Newton et al., 2013 A [29]	Prospective study	Thoracoscopic anterior spinal fusion	USA	55	14.5 ± 2	84/16%
Newton et al., 2013 B [29]	Prospective study	Open anterior spinal fusion	USA	17		
Newton et al., 2013 C [29]	Prospective study	Posterior spinal fusion	USA	64		
Garbala et al., 2023 SG [22]	Retrospective study	HGT	Poland	88	14.3 (2.8)	88.6/11.4%
Garbala et al., 2023 MG [22]	Retrospective study	HGT	Poland	107	15 (2.6)	86.9/13.1%
Yildirim et al., 2019 A [38]	RCT	Core stabilization exercises, plus structured and coached recreational activities plus usual care	Turkey	25	16.68 ± 0.74	0/100%
Yildirim et al., 2019 B [38]	RCT	Structured, coached recreational activities plus usual care	Turkey	24	16.79 ± 0.58	0/100%
Akazawa et al., 2018 [16]	Cohort	Posterior Spinal Fusion with Thoracoplasty	Japan	24	15.5 ± 2.0	95.3/4.7%
Zhou et al., 2022 [37]	Non-randomized	Halo gravity traction (HGT)	China	47	29.5 (5.8)	66/34%
Kim et al., 2008 A [14]	Prospective study	Thoracotomy	USA	64	14.9 (2.3)	90.8/9.2%
Kim et al., 2008 B [14]	Prospective study	Thoracoabdominal Approach	USA	55	15.2 (1.8)	
Byun et al., 2020 [18]	Cohort	Posterior spinal fusion	Japan	35	14.9 ± 2.0	80/20%

**Table 2:** Summary of findings of included studies.

Study	Treatment	FVC	FEV1	PEF	Other Outcomes
Kumar, 2024 A [12]	Breathing exercise protocol	FVC increased by 1.59 L from baseline	FEV1 improved by 1.48 L	PEF increased by 3.77 L per second	FEV1/FVC ratio changed by 85.12%; VC improved by 1.78 L
Kumar, 2024 B [12]	Conventional exercise therapy	FVC increased by 1.81 L from baseline	FEV1 improved by 1.66 L	PEF increased by 4.11 L per second	FEV1/FVC ratio changed by 81.32%; VC improved by 2.06 L
Mallepally et al., 2018 [28]	Posterior instrumentation and posterior spinal fusion	Preop: 90.6; Postop: 86.2 (2-year follow-up)	Preop: 86.67; Postop: 85 (2-year follow-up)	_	TLC: Preop 97.8, Postop 100 (2-year); SRS-30 Scores: 1-year 93 ± 17, 2-year 98 ± 22
Rafferty et al., 2020 [32]	Surgery	AIS cohort: 2.6 ± 0.5 L vs. 3.3 ± 0.5 L (*p* < 0.001)	2.8 ± 0.6 L vs. 3.3 ± 0.5 L (*p* < 0.001)	_	-
Ogonowska-Slodownik et al., 2021 A [30]	Aquatic breathing program	FVCex: Increased from 3.54 L to 3.61 L	FEV1: Increased from 2.88 L to 3.05 L	_	FEV1%VC: Increased from 81.84% to 86.57%; FEV1%VC%pred: Increased from 95.69% to 101.77%; MEF25 increased from 2.07 L to 2.30 L
Ogonowska-Slodownik et al., 2021 B [30]	Corrective swimming	FVCex: Decreased from 3.51 L to 3.32 L	FEV1: Decreased from 2.98 L to 2.78 L	_	FEV1%VC: Decreased from 81.39% to 81.22%; MEF25 decreased from 2.13 L to 2.04 L
Lonner et al., 2009 A [25]	Thoracotomy	Preop: 2.96 L; Follow-up: 2.70 L, Change −0.26 L (*p* < 0.001)	Preop: 2.48 L; Follow-up: 2.36 L, Change −0.12 L (*p* = 0.004)	_	TLC: Preop: 3.71 L; Follow-up: 3.71 L (no significant change)
Lonner et al., 2009 B [25]	Thoracoscopic	Preop: 2.88 L; Follow-up: 2.90 L, Change +0.02 L (NS)	Preop: 2.49 L; Follow-up: 2.53 L, Change +0.04 L (NS)	_	TLC: Preop: 3.46 L; Follow-up: 3.90 L, Change +0.44 L (*p* < 0.001, significant)
Lonner et al., 2009 C [25]	Thoracoabdominal	Preop: 3.26 L; Follow-up: 3.20 L, Change −0.06 L (NS)	Preop: 2.84 L; Follow-up: 2.81 L, Change −0.03 L (NS)	_	TLC: Preop: 4.25 L; Follow-up: 4.48 L, Change +0.23 L
Yaszay et al., 2019 [35]	Surgery	Preop: 2.98 ± 0.7 L; 5-year: 3.15 ± 0.7 L (*p* = 0.008)	Preop: 2.48 ± 0.6 L; 5-year: 2.58 ± 0.6 L (*p* = 0.10)	_	FVC% Predicted: Preop 87 ± 16%; 5-year 81 ± 13% (*p* = 0.004 significant)
Ran et al., 2014 [31]	Anterior thoracoscopic release + posterior correction	Preop: 2.47 ± 0.33 L; 2 years: 3.21 ± 1.18 L	_	_	FRC: Preop 2.86 ± 0.47; 2 years: 2.68 ± 0.90; RV: Preop 1.22 ± 0.39; 2 years: 1.13 ± 0.38; TLC: Preop 3.68 ± 0.36; 2 years: 4.32 ± 1.41
Yu et al., 2017 [36]	Thoracoscopic anterior spinal fusion	TLC: Preop: 2056.8 ± 388.4 mL; Postop: 2228.5 ± 368.9 mL (*p* = 0.01)	_	_	Right Lung Volume: Preop: 1126.3 ± 221.0 mL; Postop: 1207.6 ± 183.2 mL (*p* = 0.09); Left Lung Volume: Preop: 930.3 ± 183.3 mL; Postop: 1020.9 ± 199.9 mL (*p* = 0.01 significant)
Küçük et al., 2024 A [13]	Spinal mobilisation + core stabilisation	_	FEV1 Pre-treatment: Mean ± S.D. = 2.33 ± 0.76; Post: 2.58 ± 0.74	PEF Pre-treatment: 243.75 ± 69.77, Post: 289.25 ± 83.92 (*p* = 0.001)	Change in FEV1: Mean Δ 0.25 ± 0.62 (*p* = 0.02); Median Δ 0.22
Küçük et al., 2024 B [13]	Core stabilisation exercises	Pre: 2.74 ± 1.07 L; Post: 2.9 ± 1.27 L	Pre: 2.37 ± 0.79 L; Post: 2.53 ± 0.96 L	Pre: 237.25 ± 87.68 L/min; Post: 276.5 ± 103.68 L/min	FEV1/FVC: Pre = 88.5 ± 11.64%, Post = 90%
Amăricăi et al., 2019 [17]	Rehabilitation programme	T1: 3.18 L; T2: 3.45 L	T1: 3.03 L; T2: 3.19 L	T1: 5.12 L/min; T2: 5.22 L/min	FEV1/FVC: T1 = 87.90%, T2 = 88.50%
Faro et al., 2005 A [20]	Thoracoscopic	Pre: 2.81 ± 0.49 L; 3 months: 2.42 ± 0.55 L; 1 year: 2.82 ± 0.52 L	Pre: 2.42 ± 0.45 L; 3 months: 2.12 ± 0.49 L; 1 year: 2.49 ± 0.44 L	_	*p* = 0.025 for FEV1, *p* = 0.001 for FVC
Faro et al., 2005 B [20]	Open thoracotomy for anterior spinal fusion	Pre: 3.09 ± 0.64 L; 3 months: 2.50 ± 0.57 L; 1 year: 2.83 ± 0.61 L	Pre: 2.59 ± 0.53 L; 3 months: 2.32 ± 0.53 L; 1 year: 2.49 ± 0.52 L	_	*p* = 0.025 for FEV1, *p* = 0.001 for FVC
Dogar et al., 2021 A [19]	Scoliosis surgery and diaphragmatic breathing and pursed lip exercises	Pre: 3.28 ± 1.00 L; 1 month: 2.73 ± 0.79 L; 6 months: 3.31 ± 0.87 L	Pre: 2.89 ± 0.86 L; 1 month: 2.47 ± 0.72 L; 6 months: 2.93 ± 0.78 L	_	FEV1/FVC ratio: Pre = 89%, 6 months = 88.8%
Dogar et al., 2021 B [19]	Scoliosis surgery	Pre: 2.57 ± 0.76 L; 1 month: 2.28 ± 0.64 L; 6 months: 2.66 ± 0.66 L	Pre: 2.30 ± 0.67 L; 1 month: 2.15 ± 0.59 L; 6 months: 2.44 ± 0.56 L	_	FEV1/FVC ratio: Pre = 88.93%, 6 months = 92.26%
Hu et al., 2015 [23]	Posterior temporary internal distraction correction and definitive posterior spinal correction with posterior pedicle screw instrumentation	Pre: 59.3% ± 11.6; After internal distraction: 68.7% ± 13.7; After final correction: 71.2% ± 8.3; Final follow-up: 73.1% ± 11.9	Pre: 61.4% ± 13.6; After internal distraction: 71.3% ± 9.3; After final correction: 76.3% ± 16.7; Final follow-up: 75.5% ± 13.8	_	FEV1/FVC: Pre = 61.4%, final follow-up = 75.5%
Tis et al., 2010 [33]	Open instrumented anterior spinal fusion	Preop: 2.68 ± 0.62; Postop: 2.62 ± 0.56	Preop: 2.28 ± 0.49; Postop: 2.31 ± 0.42	_	% Predicted FEV1: Preop 75.5 ± 13; Postop 68.8 ± 12 (↓ significant); % Predicted FVC: Preop 81.6 ± 16; Postop 69.8 ± 13 (↓ significant)
Fu et al., 2015 [21]	Posterior spinal fusion surgery	_	_	_	LLV, RLV, TLV, CCLVR showed no change; LLH, RLH (↑ significant); PCSA, CCPCSAR (↓ significant)
Jeans et al., 2017 [24]	Spinal fusion	Preop: 2.8 ± 0.6; Postop: 3.0 ± 0.6 (↑)	Preop: 2.3 ± 0.4; Postop: 2.5 ± 0.5 (↑)	_	FVC%: No change; FEV1%: No significant change
Min et al., 2012 [27]	Anterior short correction	Preop: 2842 mL; Last: 2812 mL (no change)	-	_	FVC%: No significant change between Preop and Last follow-up
Abd El Ghafar et al., 2022 A [15]	Hippotherapy + Schroth exercises	Preop: 2.62 ± 0.49; Postop: 2.97 ± 0.57 (↑)	Preop: 2.28 ± 0.44; Postop: 2.64 ± 0.58 (↑)	_	MVV: ↑ significant; FEV1/FVC: ↑ significant
Abd El Ghafar et al., 2022 B [15]	Schroth exercises	Preop: 2.47 ± 0.45; Postop: 2.65 ± 0.59 (↑)	Preop: 2.31 ± 0.41; Postop: 2.56 ± 0.58 (↑)	_	MVV: ↑ significant; FEV1/FVC: ↑ significant
Vitale et al., 2008 [34]	Spinal fusion	FVC: 74.4 ± 19.4 (↓)	FEV1: 73.0 ± 20.2 (↓)	_	TLC: 88.5 ± 17.0 (↓); VC: 75.6 ± 19.6 (↓)
Laurentowska et al., 2009 [26]	Cotrel-Dubousset + Rehab program	VC: 3.05 ± 0.439	FEV1%: 92.18 ± 5.002	_	% Predicted FEV1: 108.27 ± 5.781; MVV: 87.60 ± 13.876; MVV%pred: 70.54 ± 13.201
Yuan et al., 2005 [39]	Surgery	Declined up to 60% post-surgery, nadir at 3 days, recovery to 70% baseline by 6 months.	Not significantly correlated with scoliosis etiology or surgery type.	_	No statistical differences in recovery trends based on scoliosis etiology or surgery type.
Newton et al., 2013 A [29]	Thoracoscopic anterior spinal fusion	Pre: 2.7 ± 0.5 L; Post: 2.7 ± 0.5 L; Change: +0.02 ± 0.5.	Pre: 2.3 ± 0.4 L; Post: 2.4 ± 0.4 L; Change: +0.1 ± 0.4.	_	TLC (%predicted) Pre: 86 ± 10; Post: 87 ± 9; Change: +1 ± 7.
Newton et al., 2013 B [29]	Open anterior spinal fusion	Pre: 2.8 ± 0.6 L; Post: 2.5 ± 0.5 L; Change: −0.2 ± 0.3.	Pre: 2.4 ± 0.5 L; Post: 2.3 ± 0.5 L; Change: −0.2 ± 0.4.	_	TLC (%predicted) Pre: 88 ± 17; Post: 73 ± 7; Change: −15 ± 13.
Newton et al., 2013 C [29]	Posterior spinal fusion	Pre: 3.1 ± 0.7 L; Post: 3.3 ± 0.7 L; Change: +0.2 ± 0.4.	Pre: 2.6 ± 0.6 L; Post: 2.7 ± 0.6 L; Change: +0.1 ± 0.4.	_	TLC (%predicted) Pre: 90 ± 13; Post: 78 ± 26; Change: −20 ± 36.
Garbala et al., 2023 SG [22]	Halo gravity traction (HGT) for severe group	Pre: 51.2 ± 12.8%; Follow-up: 69.9 ± 11.2% (*p* < 0.001).	Pre: 60.8 ± 13.9%; Follow-up: 76.9 ± 14.5% (*p* < 0.001).	_	Improved pulmonary function observed for severe group.
Garbala et al., 2023 MG [22]	Halo gravity traction (HGT) for moderate group	Pre: 83 ± 11.2%; Follow-up: 79 ± 13.2% (*p* = 0.12).	Pre: 77 ± 12.8%; Follow-up: 81 ± 12.8% (*p* = 0.09).	_	Minimal changes in pulmonary function in moderate group.
Yildirim et al., 2019 A [38]	Core stabilization exercises + recreational activities + usual care	Baseline: 85.70 ± 11.62%; Post: 91.51 ± 13.22%; Change: +5.8 (*p* < 0.0001).	Baseline: 92.74 ± 13.18%; Post: 100.08 ± 13.77%; Change: +7.35 (*p* < 0.0001).	Baseline: 75.00 ± 18.34%; Post: 88.32 ± 15.03%; Change: +13.32 (*p* < 0.0001).	Improved FEF25–75% from 97.68 ± 19.66% to 109.52 ± 18.20% (*p* = 0.0002).
Yildirim et al., 2019 B [38]	Recreational activities + usual care	Baseline: 88.29 ± 13.29%; Post: 89.25 ± 12.55%; Change: +0.96 (*p* = 0.064).	Baseline: 95.00 ± 11.56%; Post: 97.91 ± 11.37%; Change: +2.92 (*p* = 0.035).	Baseline: 78.79 ± 17.77%; Post: 83.45 ± 19.62%; Change: +4.67 (*p* < 0.0001).	Improved FEF25–75% from 98.88 ± 19.37% to 105.12 ± 14.09% (*p* = 0.015).
Akazawa et al., 2018 [16]	Posterior spinal fusion with thoracoplasty	Pre: 2.27 ± 0.64 L; Final: 2.38 ± 0.50 L; Change: +0.11 (*p* = 0.240).	Pre: 1.88 ± 0.52 L; Final: 2.05 ± 0.42 L; Change: +0.17 (*p* = 0.045).	Pre: 3.67 ± 1.51 L/s; Final: 4.38 ± 1.18 L/s; Change: +0.71 (*p* = 0.029).	Significant improvements in %FEV1 (*p* = 0.001), FEV1/FVC (*p* = 0.019), and other flow measures like V25 and V50.
Zhou et al., 2022 [37]	Halo gravity traction (HGT)	- Pre-traction: 2.50 (0.98) - Post-traction: 2.08 (0.75) - Postoperation: 2.45 (0.81) - 2-year follow-up: 2.45 (0.83) - FVC%: Pre-traction: 63.7 (13.9), Post-traction: 60.5 (14.2), 2-year follow-up: 64.1 (15.2)	- Pre-traction: 1.93 (0.80) - Post-traction: 1.63 (0.59) Postoperation: 2.17 (0.55) - 2-year follow-up: 2.28 (0.70) - FEV1%: Pre-traction: 66.6 (18.2), Post-traction: 58.7 (16.3), 2-year follow-up: 69.8 (11.4)	_	- Total lung volume: Pre-traction: 860.8 (36.9), 5-year follow-up: 1095.9 (59.4) - Concave lung volume: Pre-traction: 327.7 (42.6), 5-year follow-up: 514.7 (46.3) - Convex lung volume: Pre-traction: 533.1 (33.3), 5-year follow-up: 581.2 (63.2)
Kim et al., 2018 A [14]	Thoracotomy	- Preoperative: 3.05 L ± 0.70 - 2-Year Postoperative: 2.74 L ± 0.61 - Difference: −0.31 L ± 0.43 (12% ± 17.2) - *p* = 0.0001	- Preoperative: 2.56 L ± 0.55 - 2-Year Postoperative: 2.35 L ± 0.49 - Difference: −0.21 L ± 0.38 (7% ± 13.1) - *p* = 0.015, 0.0001	_	- FVC% predictive value: Preoperative: 87% ± 13.9, Postoperative: 74% ± 12.2 - *p* = 0.01, 0.045 (difference)
Kim et al., 2018 B [14]	Thoracoabdominal Approach	- Preoperative: 3.27 L ± 0.67 - 2-Year Postoperative: 3.21 L ± 0.64 - Difference: −0.06 L ± 0.32 (2% ± 9.7) - *p* = 0.08 (preop), 0.0001 (postop)	- Preoperative: 2.82 L ± 0.60 - 2-Year Postoperative: 2.81 L ± 0.52 - Difference: −0.02 L ± 0.29 (0% ± 10.5) - *p* = 0.015, 0.0001	_	- FVC% predictive value: Preoperative: 95% ± 18.2, Postoperative: 87% ± 14.6 - *p* = 0.01, 0.002 (difference)
Byun et al., 2020 [18]	Posterior spinal fusion	- Preoperative: 2.3 ± 0.7 (0.6–3.8) - Final Follow-Up: 2.5 ± 0.6 (1.0–3.9) - *p* = 0.22	- Preoperative: 2.0 ± 0.6 (0.6–3.5) - Final Follow-Up: 2.2 ± 0.5 (1.3–3.4) - *p* = 0.08	_	- Predicted FVC: Preoperative: 3.1 ± 0.5, Final Follow-Up: 3.3 ± 0.4 - %FEV1: Preoperative: 91.8 ± 42.6, Final Follow-Up: 82.8 ± 12.5

**Table 3:** Cochrane risk-of-bias assessment.

Study	Random Sequence Generation	Allocation Concealment	Blinding of Participants and Personnel	Blinding of Outcome Assessment	Incomplete Outcome Data	Selective Reporting	Other Bias
Küçük et al., 2024 [13]	Low	Low	Low	Low	Low	Low	Low
Doger et al., 2021 [19]	Low	Unclear	Unclear	Unclear	Low	Low	Low
Abdel Ghafar et al., 2022 [15]	Low	Low	Low	High	High	Low	Low
Yildirim et al., 2019 [38]	Low	Unclear	Unclear	Unclear	High	Low	Low

**Table 4:** NOS risk-of-bias assessment.

Studies	Selection	Comparability	Exposure/Outcome	Overall Star Rating
Kumar, 2024 [12]	**	**	*	5
Mallepally et al., 2018 [28]	***	*	**	6
Rafferty et al., 2020 [32]	**	**	***	7
Ogonowska-Slodownik et al., 2020 [30]	***	**	*	6
Lonner et al., 2009 [25]	****	**	**	8
Yaszay et al., 2019 [35]	***	*	**	6
Ran et al., 2019 [31]	***	**	*	6
Yu et al., 2017 [36]	**	*	***	6
Amăricăi et al., 2019 [17]	**	*	**	5
Faro et al., 2005 [20]	**	*	**	5
Hu et al., 2015 [23]	*	**	*	4
Tis et al., 2010 [33]	***	**	***	8
Fu et al., 2015 [21]	**	**		4
Jeans et al., 2017 [24]	***	**	**	7
Min et al., 2012 [27]	**	**	**	6
Vitale et al., 2008 [34]	***	**	**	7
Laurentowska et al., 2009 [26]	**	*	*	4
Yuan et al., 2005 [39]	***	*	**	6
Newton et al., 2013 [29]	****	**	**	8
Grabala et al., 2023 [22]	***	**	***	8
Akazawa et al., 2018 [16]	***	*	***	7
Zhou et al., 2022 [37]	***	*	***	7
Kim et al., 2008 [14]	****	**	**	8
Byun et al., 2020 [18]	***	**	**	7

* Point, ** two points, *** three points, **** four points.

## Data Availability

Data are available when requested from the author.

## Supplementary Materials

The following supporting information can be downloaded at: https://www.mdpi.com/article/10.3390/medicina61071127/s1, PRISMA_2020_checklist. Reference [40] are cited in the Supplementary Materials.

## Abbreviations

The following abbreviations are used in this manuscript:

DOAJ	Directory of open access journals
TLA	Three letter acronym
LD	Linear dichroism

## References

[1] Rolton D., Nnadi C., Fairbank J. (2014). Scoliosis: A review. Paediatr. Child Health.

[2] Karol L.A. (2019). The Natural History of Early-onset Scoliosis. J. Pediatr. Orthop..

[3] Behr J., Furst D.E. (2008). Pulmonary function tests. Rheumatology.

[4] Ruppel G.L., Enright P.L. (2012). Pulmonary Function Testing. Respir. Care.

[5] Tsiligiannis T., Grivas T. (2012). Pulmonary function in children with idiopathic scoliosis. Scoliosis.

[6] Zhang J.G., Wang W., Qiu G.X., Wang Y.P., Weng X.S., Xu H.G. (2005). The Role of Preoperative Pulmonary Function Tests in the Surgical Treatment of Scoliosis. Spine.

[7] Newton P.O., Faro F.D., Gollogly S., Betz R.R., Lenke L.G., Lowe T.G. (2005). Results of Preoperative Pulmonary Function Testing of Adolescents with Idiopathic Scoliosis: A Study of Six Hundred and Thirty-one Patients. JBJS.

[8] Newton P.O., Perry A., Bastrom T.P., Lenke L.G., Betz R.R., Clements D., D’Andrea L. (2007). Predictors of Change in Postoperative Pulmonary Function in Adolescent Idiopathic Scoliosis: A Prospective Study of 254 Patients. Spine.

[9] Pehrsson K., Bake B., Larsson S., Nachemson A. (1991). Lung function in adult idiopathic scoliosis: A 20 year follow up. Thorax.

[10] Kim Y.J., Lenke L.G., Bridwell K.H., Kim K.L., Steger-May K. (2005). Pulmonary Function in Adolescent Idiopathic Scoliosis Relative to the Surgical Procedure. JBJS.

[11] Julian P.T.H., Douglas G.A., Peter C.G., Peter J., David M., Andrew D.O., Jelena S., Kenneth F.S., Laura W., Jonathan A.C.S. (2011). The Cochrane Collaboration’s tool for assessing risk of bias in randomised trials. BMJ.

[12] Kumar A. (2024). Assessing the Impact of Rotational Angular Breathing on Lung Functions and Health-Related Quality of Life in Adolescent Idiopathic Scoliosis. Int. J. Physiother..

[13] Küçük E., Öten E., Coskun G. (2024). Effects of spinal mobilisation in adolescent idiopathic scoliosis: A randomised controlled trial. J. Paediatr. Child Health.

[14] Kim Y.J., Lenke L.G., Bridwell K.H., Cheh G., Sides B., Whorton J. (2008). Prospective pulmonary function comparison of anterior spinal fusion in adolescent idiopathic scoliosis: Thoracotomy versus thoracoabdominal approach. Spine.

[15] Abdel Ghafar M.A., Abdelraouf O.R., Abdel-Aziem A.A., Elnegamy T.E., Mohamed M.E., Yehia A.M., Samir Mousa G. (2022). Pulmonary function and aerobic capacity responses to equine-assisted therapy in adolescents with idiopathic scoliosis: A randomized controlled trial. J. Rehabil. Med..

[16] Akazawa T., Kuroya S., Iinuma M., Asano K., Torii Y., Umehara T., Kotani T., Sakuma T., Minami S., Orita S. (2018). Pulmonary function and thoracic deformities in adolescent idiopathic scoliosis 27 years or longer after spinal fusion with Harrington instrument. J. Orthop. Sci..

[17] Amăricăi E., Suciu O., Onofrei R.R., Miclaus R.S., Iacob R.E., Catan L., Popoiu C.M., Cerbu S., Boia E. (2020). Respiratory function, functional capacity, and physical activity behaviours in children and adolescents with scoliosis. J. Int. Med. Res..

[18] Byun Y.M., Iida T., Yamada K., Abumi K., Kokabu T., Iwata A., Iwasaki N., Sudo H. (2020). Long-term pulmonary function after posterior spinal fusion in main thoracic adolescent idiopathic scoliosis. PLoS ONE.

[19] Dogar F., Argun M., Erdem S., Gurbuz K., Argun A.S., Kafadar I.H. (2021). Clinical and radiological results of surgically treated patients with adolescent idiopathic scoliosis and the effects of pulmonary rehabilitation on respiration functions. Medicine.

[20] Faro F.D., Marks M.C., Newton P.O., Blanke K., Lenke L.G. (2005). Perioperative changes in pulmonary function after anterior scoliosis instrumentation: Thoracoscopic versus open approaches. Spine.

[21] Fu J., Liu C., Zhang Y.G., Zheng G.Q., Zhang G.Y., Song K., Tang X.Y., Wang Y. (2015). Three-dimensional Computed Tomography for Assessing Lung Morphology in Adolescent Idiopathic Scoliosis following Posterior Spinal Fusion Surgery. Orthop. Surg..

[22] Grabala P., Helenius I.J., Buchowski J.M., Shah S.A. (2023). The Efficacy of a Posterior Approach to Surgical Correction for Neglected Idiopathic Scoliosis: A Comparative Analysis According to Health-Related Quality of Life, Pulmonary Function, Back Pain and Sexual Function. Children.

[23] Hu H.M., Hui H., Zhang H.P., Huang D.G., Liu Z.K., Zhao Y.T., He S.M., Zhang X.F., He B.R., Hao D.J. (2016). The impact of posterior temporary internal distraction on stepwise corrective surgery for extremely severe and rigid scoliosis greater than 130°. Eur. Spine J..

[24] Jeans K.A., Lovejoy J.F., Karol L.A., McClung A.M. (2017). How Is Pulmonary Function and Exercise Tolerance Affected in Patients With AIS Who Have Undergone Spinal Fusion?. Spine Deform..

[25] Lonner B.S., Auerbach J.D., Estreicher M.B., Betz R.R., Crawford A.H., Lenke L.G., Newton P.O. (2009). Pulmonary function changes after various anterior approaches in the treatment of adolescent idiopathic scoliosis. J. Spinal Disord. Tech..

[26] Laurentowska M., Glowacki M., Michalak E., Deskur-Śmielecka E., Barinow-Wojewódzki A. (2009). Effects of rehabilitation based on endurance training in adolescent ghtls with surgically treated scoliosis. Biol. Sport.

[27] Min K., Haefeli M., Mueller D., Klammer G., Hahn F. (2012). Anterior short correction in thoracic adolescent idiopathic scoliosis with mini-open thoracotomy approach: Prospective clinical, radiological and pulmonary function results. Eur. Spine J..

[28] Mallepally A.R., Iyengar R.S., Patnala C.S. (2018). Analysis of Adolescent Idiopathic Thoracic Scoliosis Treated with Posterior Instrumentation and Fusion: Our Experience. J. Clin. Diagn. Res..

[29] Newton P.O., Marks M.C., Bastrom T.P., Betz R., Clements D., Lonner B., Crawford A., Shufflebarger H., O’Brien M., Yaszay B. (2013). Surgical Treatment of Lenke 1 Main Thoracic Idiopathic Scoliosis Results of a Prospective, Multicenter Study. Spine.

[30] Ogonowska-Slodownik A., Kaczmarczyk K., Kokowicz G., Morgulec-Adamowicz N. (2020). Does the Aquatic Breathing Program Improve Lung Function in Adolescents with Scoliosis?. Phys. Occup. Ther. Pediatr..

[31] Ran B., Li Q., Li C., Li M., Chen J.Y., Wang L.X., Qiao Y.H., Guan J.H., Wang Z.W. (2014). Effect of Anterior Thoracoscopic Release Combined with the Posterior Correction Operation on the Pulmonary Function of Patients with Idiopathic Scoliosis. Thorac. Cardiovasc. Surg..

[32] Rafferty A., Donne B., Kiely P., Fleming N. (2020). Functional deficits in post-operative adolescent idiopathic scoliosis. Physiother. Pract. Res..

[33] Tis J.E., O’Brien M.F., Newton P.O., Lenke L.G., Clements D.H., Harms J., Betz R.R. (2010). Adolescent idiopathic scoliosis treated with open instrumented anterior spinal fusion: Five-year follow-up. Spine.

[34] Vitale M.G., Matsumoto H., Bye M.R., Gomez J.A., Booker W.A., Hyman J.E., Roye Jr D.P. (2008). A retrospective cohort study of pulmonary function, radiographic measures, and quality of life in children with congenital scoliosis: An evaluation of patient outcomes after early spinal fusion. Spine.

[35] Yaszay B., Jankowski P.P., Bastrom T.P., Lonner B., Betz R., Shah S., Asghar J., Miyanji F., Samdani A., Newton P.O. (2019). Progressive decline in pulmonary function 5 years post-operatively in patients who underwent anterior instrumentation for surgical correction of adolescent idiopathic scoliosis. Eur. Spine J..

[36] Yu C.G., Grant C.A., Izatt M.T., Labrom R.D., Askin G.N., Adam C.J., Little J.P. (2017). Change in Lung Volume Following Thoracoscopic Anterior Spinal Fusion Surgery. Spine.

[37] Zhou X., Li X., Wu Q., Liang J., Guo H., Jin M., Zhu X., Du Q. (2022). Three-dimensional corrective exercise therapy for idiopathic scoliosis: Study protocol for a prospective non-randomized trial. BMC Musculoskelet. Disord..

[38] Yildirim S., Ozyilmaz S., Elmadag N.M., Yabaci A. (2022). Effects of Core Stabilization Exercises on Pulmonary Function, Respiratory Muscle Strength, Peripheral Muscle Strength, Functional Capacity, and Perceived Appearance in Children With Adolescent Idiopathic Scoliosis: A Randomized Controlled Trial. Am. J. Phys. Med. Rehabil..

[39] Yuan N., Fraire J.A., Margetis M.M., Skaggs D.L., Tolo V.T., Keens T.G. (2005). The effect of scoliosis surgery on lung function in the immediate postoperative period. Spine.

[40] Page M.J., McKenzie J.E., Bossuyt P.M., Boutron I., Hoffmann T.C., Mulrow C.D., Shamseer L., Tetzlaff J.M., Akl E.A., Brennan S.E. (2021). The PRISMA 2020 statement: An updated guideline for reporting systematic reviews. BMJ.

